# A Predictive Model Combining Fecal Calgranulin B and Fecal Occult Blood Tests Can Improve the Diagnosis of Colorectal Cancer

**DOI:** 10.1371/journal.pone.0106182

**Published:** 2014-09-04

**Authors:** Byung Chang Kim, Jungnam Joo, Hee Jin Chang, Hyun Yang Yeo, Byong Chul Yoo, Boram Park, Ji Won Park, Dae Kyung Sohn, Chang Won Hong, Kyung Su Han

**Affiliations:** 1 Center for Colorectal Cancer, National Cancer Center, Ilsan-gu, Goyang, Gyeonggi, Korea; 2 Center for Cancer Prevention and Detection, National Cancer Center, Ilsan-gu, Goyang, Gyeonggi, Korea; 3 Biometric Research Branch, National Cancer Center, Ilsan-gu, Goyang, Gyeonggi, Korea; 4 Research Institute and Hospital, National Cancer Center, Ilsan-gu, Goyang, Gyeonggi, Korea; University General Hospital of Heraklion and Laboratory of Tumor Cell Biology, School of Medicine, University of Crete, Greece

## Abstract

**Aim:**

Current fecal screening tools for colorectal cancer (CRC), such as fecal occult blood tests (FOBT), are limited by their low sensitivity. Calgranulin B (CALB) was previously reported as a candidate fecal marker for CRC. This study investigated whether a combination of the FOBT and fecal CALB has increased sensitivity and specificity for a diagnosis of CRC.

**Materials and Methods:**

Patients with CRC (*n* = 175), and healthy individuals (controls; *n* = 151) were enrolled into the development (81 cases and 51 controls) and validation (94 cases and 100 controls) sets. Stool samples were collected before bowel preparation. CALB levels were determined by western blotting. FOBT and fecal CALB results were used to develop a predictive model based on logistic regression analysis. The benefit of adding CALB to a model with only FOBT was evaluated as an increased area under the receiver operating curve (AUC), partial AUC, and reclassification improvement (RI) in cases and controls, and net reclassification improvement (NRI).

**Results:**

Mean CALB level was significantly higher in CRC patients than in controls (*P*<0.001). CALB was not associated with tumor stage or cancer site, but positivity on the FOBT was significantly higher in advanced than in earlier tumor stages. At a specificity of 90%, the cross-validated AUC and sensitivity were 89.81% and 82.72%, respectively, in the development set, and 92.74% and 79.79%, respectively, in the validation set. The incremental benefit of adding CALB to the model, as shown by the increase in AUC, had a p-value of 0.0499. RI in cases and controls and NRI all revealed that adding CALB significantly improved the prediction model.

**Conclusion:**

A predictive model using a combination of FOBT and CALB may have greater sensitivity and specificity and AUC for predicting CRC than models using a single marker.

## Introduction

Colorectal cancer (CRC) is the third most common malignancy worldwide [1], and its prevalence in Korea is increasing dramatically [2]. Like other cancers, the survival of patients with CRC is closely related to the stage at diagnosis. Early detection of CRC is not only associated with improved outcomes [3], but also significantly reduces the cost of treatment. Current screening tests for CRC involve the detection of blood in stool samples and the visualization of gross abnormalities by colonoscopy. Although colonoscopy is still the gold standard method for CRC screening, diagnosis and treatment, it is invasive and associated with poor patient acceptability and high cost. In contrast, stool tests are non-invasive, do not require bowel preparation, may represent the entire colon, and are suitable for mass screening, and the specimens are easy to transport [1].

Stool markers are currently classified as those that leak through, are secreted by, or are shed from neoplastic cells [4], [5]. Hemoglobin is a leaked protein measured in the conventional fecal occult blood test (FOBT), which is commonly used in large-scale CRC screening programs [1], [6]–[10]. Calprotectin is another leaked protein that may be a marker for CRC [7], [9], [11]. These markers, however, have relatively poor sensitivity and specificity. Indeed, there are still no non-invasive screening tools that show high sensitivity and high specificity for CRC.

Elevated levels of calgranulin B (CALB) have been detected in stool samples from CRC patients [7]. Calprotectin is a heterodimer composed of calgranulin A and CALB connected by a peptide bridge. CALB showed higher sensitivity but lower specificity for CRC than FOBT [7]. We hypothesized that a combination of candidate fecal markers, rather than a single marker, would improve the diagnosis of CRC. We therefore prospectively investigated whether the combination of FOBT and CALB improved the sensitivity and specificity of either alone in diagnosing CRC.

## Materials and Methods

### Study design

Subjects were divided into two independent sets, a development and a validation set. The development cohort consisted of patients from our previous study [7], in which we first performed western blot analysis of CALB, followed by ELISA analysis. Western blot analysis was performed on samples from 81 patients with CRC. The development set of this study included these 81 CRC patients and 51 controls. Since we found interesting results by adding CALB data from western blot analysis to FOBT, we attempted to validate the model in an independent patient cohort. The sample size for the validation set was based on previous results [12], which showed that 85 case subjects would be needed for an expected sensitivity of 85% and to yield a 95% probability that the estimated 95% lower confidence limit of sensitivity would be above 75%. Estimating that the drop-out rate, due, for example, to sample failure, would be 10%, we estimated that 94 case subjects, independent of the development cohort, would be required. None of these patients dropped out, however, and all 94 subjects were included in the validation set. A similar calculation was performed for control subjects, but slightly more were processed due to greater availability. Consequently, 100 control subjects independent of the development cohort were included in the validation set. Based on 94 subjects, we calculated that, at a specificity of 90%, the estimated 95% lower confidence interval for specificity would be above 75% and 80% in 99% and 85% of subjects, respectively. The addition of six additional subjects would increase these probabilities to 99.5% and 87.3%, respectively.

### Subjects and stool samples

Subjects were divided into a development set and a validation set. The development set included 81 patients diagnosed with CRC and 51 controls, of mean (SD) age 63.16 (10.42) years and 50.24 (10.12) years, respectively. The validation set included 94 patients with CRC and 100 controls, of mean (SD) age 62.96 (11.97) years and 49.43 (10.78) years, respectively.

CRCs were diagnosed by colonoscopy and histopathology. All histopathological examinations were performed by a single gastrointestinal pathologist (HJ Chang) and the results were classified according to World Health Organization guidelines, with carcinomas classified by American Joint Committee on Cancer stage [13], [14]. Lesions were also classified according to their location in the right or left side of the colon. The right colon was defined as extending from the cecum to the splenic flexus, while the left colon was defined as extending from the descending colon to the rectum [15]. All subjects in the control group had negative findings on colonoscopy.

All enrolled subjects underwent colonoscopy, with preparation and sedation depending on subject characteristics. Stool samples were collected before bowel preparation.

We provided written informed consent and explained information of the study to participants by research coordinators before they participated this study. After they completely understood and agreed to this study, they signed an informed consent form. The research protocols for the present study were reviewed and approved by the Institutional Review Board of the National Cancer Center, Korea (NCCNTS-08-354).

### Stool sample preparation

Stool samples (0.1 g in 0.3 ml PBS) containing protease inhibitors were vortexed and centrifuged at 12,000×g for 10 min. The supernatants containing extracted proteins were collected without disturbing the pellets and used for western blotting.

### Western blotting

Equivalent amounts of stool protein (10 µg) were subjected to SDS-PAGE and transferred to PVDF membranes (Millipore, Billerica, MA). The membranes were incubated for 2 h at 4°C in 1% Tween 20-TBS containing 1.5% non-fat dried milk (Bio-Rad, Berkeley, CA) and 1 mM MgCl_2_ to block non-specific binding, and subsequently were incubated for 2 h at room temperature with primary antibodies against CALB (both from Santa Cruz Biotechnology, Santa Cruz, CA). After three washes for 15 min each with blocking solution, the membranes were incubated with diluted HRP-conjugated secondary antibody (Southern Biotech, Birmingham, UK) for 1 h at room temperature. The membranes were again washed three times for 15 min each with blocking solution, incubated with WEST-ZOL® plus chemiluminescence reagent (iNtRON Biotechnology, Gyeonggi, Korea) for 1 min, and exposed to film (Kodak Blue XB-1; Kodak, Rochester, NY). The optical density (arbitrary unit) of CALB signals was measured by *TINA 2.10e* software (Raytest Isotopenmessgeraete GmBH, Straubenhardt, Germany), and the relative level of CALB in stool was quantified by comparing its level of expression in stool samples to that in the human breast cancer cell line SK-BR-3 (10 µg).

### FOBT

FOBT was performed using an OC-sensor kit (EIKEN Chemical Co. Ltd., Tokyo, Japan), according to the manufacturer’s instructions, by researchers blinded to the source of each sample. The FOBT used in this study did not require dietary restrictions. The analytical cut-off for FOBT positivity was 100 ng Hb/ml.

### Statistical analysis

Between group levels of CALB were tested using non-parametric methods (Wilcoxon rank-sum test and Kruskal-Wallis test). The proportion of samples positive for FOBT in two groups was compared using Pearson’s chi-square test. The CRC predictive model was developed based on logistic regression, which estimates the probability of CRC based on exploratory variables. To accommodate the non-normality of CALB measurements, their rank was used in the logistic regression analysis as a covariate [16].

Two prediction models were considered. The first model used only FOBT, and the second included both FOBT and CALB. Because of the imbalances in age between CRC patients and controls in both the development and validation sets, age was adjusted for in both models. The ability of these models to perform in an independent cohort was assessed by receiver operating curve (ROC) analysis; the areas under the ROC curves (AUC), and the partial areas under the curve (pAUC) corresponding to a specificity >0.9 were first validated internally using the leave-one-out cross validation (LOOCV) technique. After internal validation, the prediction models built using the development set was applied to the validation set, and the performances of the models were assessed externally. Once both the internal and external validations revealed acceptable performance, the final predictive model for use in future subjects was developed using the total data set, comprised of both the development and validation sets, because the accuracy in estimating the effects of risk factors increases with increasing sample size [17]. Schematics of these model development procedures are shown in Figure S1.

The incremental benefit of a new marker, CALB, was assessed by determining increases in AUC and pAUC, reclassification improvements (RI) for cases and controls, and net-reclassification improvements (NRI) [18]. The AUC measures how well the model distinguishes between CRC patients and controls, and it can be interpreted as the likelihood that a model will assign higher probability to a CRC patient than to a control subject. The pAUC only considers ROCs corresponding to preset values of sensitivity or specificity; in this study, specificities >0.9 were considered, making 10% the maximum achievable value. Statistically significant increases in AUC and pAUC, however, are difficult to determine for predictive models with reasonably good performance. NRI is an alternative measure proposed to overcome this problem [18]. To measure NRI, RI is first calculated separately for the patient and control groups. RI in CRC patients was defined as the proportion of subjects whose estimated probability of an event is higher with the newer than the older model minus the proportion of subjects whose estimated probability is lower. RI in control subjects was defined as the proportion of subjects whose estimated probability is lower minus the proportion of subjects whose estimated probability is higher. The sum of these two measures is the NRI, with an asymptotic distribution used to evaluate its significance [18]. All statistical analyses were performed using R statistical software version 2.15.2. (http://www.r-project.org).

## Results

### Fecal CALB and FOBT

**Figure 1:**
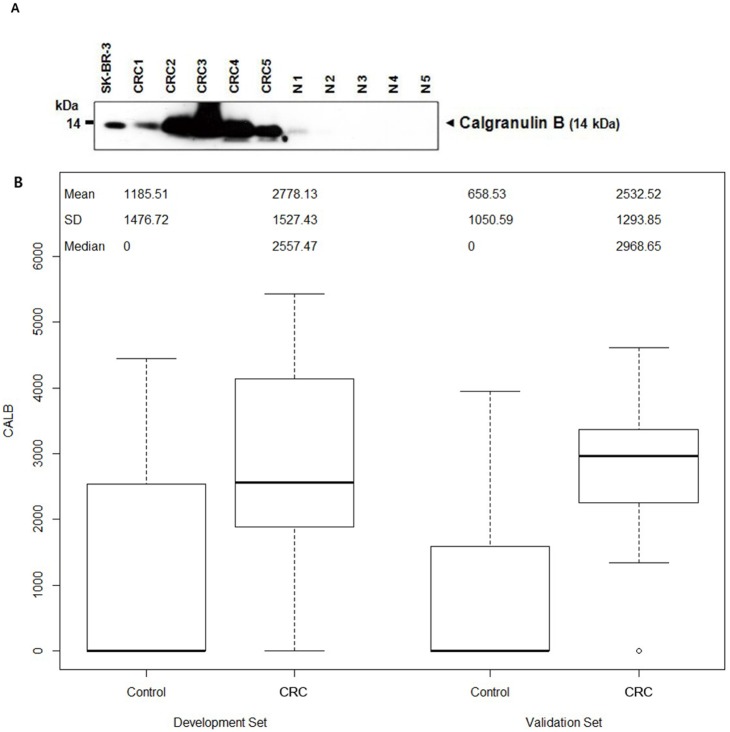
Calgranulin B level in stool of cancer and control patients (A) Western blotting showing various levels of calgranulin B in stool samples from CRC patients (CRC1–CRC5) and healthy controls (N1–N5). The human breast cancer cell line SK-BR-3 was used as a positive control for calgranulin B. (**B**) CALB concentrations in case (colorectal cancer patients) and control patients are shown separately for the development and validation sets.

Median CALB concentration was significantly higher in stool samples from CRC patients than in those from healthy controls (*P*<0.001) (Figure 1A, 1B and Table 1). Thus, fecal CALB alone may distinguish between CRC patients and healthy individuals with high probability.

**Table 1:** Expression level (optical density of immunoreactive signal; arbitrary unit) of calgranulin B and positivity rate of FOBT according to the type of colorectal disease.

		Calgranulin B			FOBT	
Development set		Median	Range	*P*-value	Positive	*P*-value
**Cancer** (*n* = 81)		2557.5	0.0–5432.1	<0.001*	42/81 (51.9%)	<.0001
** T stage**	**T1 (n = 14)**	1813.2	0.0–4804.9	0.314	1/14 (7.1%)	0.001
	**T2 (n = 10)**	2998	0.0–5181.2		6/10 (60.0%)	
	**T3 (n = 47)**	2557.5	0.0–5432.1		26/47 (55.3%)	
	**T4 (n = 10)**	3112.2	1507.3–4926.3		9/10 (90.0%)	
** Site**						
	**Right (n = 21)**	2566.7	0.0–5288.8	0.863	11/21 (52.4%)	0.955
	**Left (n = 60)**	2522.5	0.0–5432.1		31/60 (51.7%)	
**Controls (** ***n*** ** = 51)**		0	0.0–4448.9	–	1/51 (1.9%)	–
**Validation set**						
**Cancer** (*n* = 94)		2968.6	0.0–4608.1	<0.001*	52/94 (55.3%)	<.0001
** T stage**	**T1 (n = 10)**	2577.5	0.0–3300.3	0.113	2/10 (20.0%)	0.019
	**T2 (n = 8)**	2329.8	0.0–3359.5		2/8 (25.0%)	
	**T3 (n = 59)**	3069.8	0.0–4608.1		37/59 (62.7%)	
	**T4 (n = 17)**	2942.4	0.0–4585.7		11/17 (64.7%)	
** Site**						
	**Right (n = 25)**	2842.2	0.0–3985.8	0.674	13/25 (52.0%)	0.697
	**Left (n = 69)**	2992.5	0.0–4608.1		39/69 (56.5%)	
**Controls (** ***n*** ** = 100)**		0	0.0–3955.3	–	0/100	–

FOBT, fecal occult blood test (100 ng/mL stool).

*by the Wilcoxon rank sum test.

Among patients with CRC, the levels of CALB were not associated with tumor stage or the site of cancer in both the development and validation sets (Table 1). However, the FOBT positivity rate was significantly higher in patients with more than less advanced tumor stages in both the development and validation sets (*P*<0.05) (Table 1).

### Performance of predictive models including fecal markers in the diagnosis of CRC

The top panel of Table 2 shows the sensitivity at a specificity closest to 90%, the AUC, and pAUC at a specificity of 90%–100% in the development set and the bias-corrected values of these measures via internal validation using LOOCV. At a specificity of 90.2%, the sensitivity of the model using FOBT alone was 75.31%, the AUC was 89.52% (95% CI 84.19%–94.85%) and the pAUC was 6.65%. At the same specificity, the sensitivity of the model that included both FOBT and CALB was 83.95%, the AUC was 92.05% (95% CI 87.59%–95.50%) and the pAUC was 7.02%, with all improved when compared with the model using only FOBT. Similarly, following bias correction via LOOCV, the sensitivity (82.72% versus 75.31%), AUC (89.81% [95% CI 84.02%–95.60%] versus 87.78%), and pAUC (5.70% versus 5.62%) were higher for the model that included both FOBT and CALB than for the model that included only FOBT. The performances of these models on the independent validation set are summarized in the bottom panel of Table 2, with all results closely matched to the estimated values after LOOCV.

**Table 2:** Cross-validated sensitivity at the specificity closest to 90% and the AUC and pAUC of the two models (all in %).

	Sensitivity*	Specificity**	AUC	Partial AUC***
Development set				
Model 1: AGE + FOBT	75.31	90.2	89.52	6.65
Model 2: AGE + FOBT + CALB	83.95	90.2	92.05	7.02
Development set (Leave-one-out cross-validation)				
Model 1: AGE + FOBT	75.31	90.2	87.78	5.62
Model 2: AGE + FOBT + CALB	82.72	90.2	89.81	5.70
Validation set				
Model 1: AGE + FOBT	79.79	90	90.65	7.34
Model 2: AGE + FOBT + CALB	79.79	90	92.74	7.71

AUC. Area under the curve; pAUC, partial area under the curve, FOBT. Fecal occult blood test; CALB, calgranulin B.

*Sensitivity when the specificity of the model developing samples is set at closest to 90%.

**Observed specificity closest to 90%.

***Partial AUC for specificities range from 90% to 100%.

**Figure 2:**
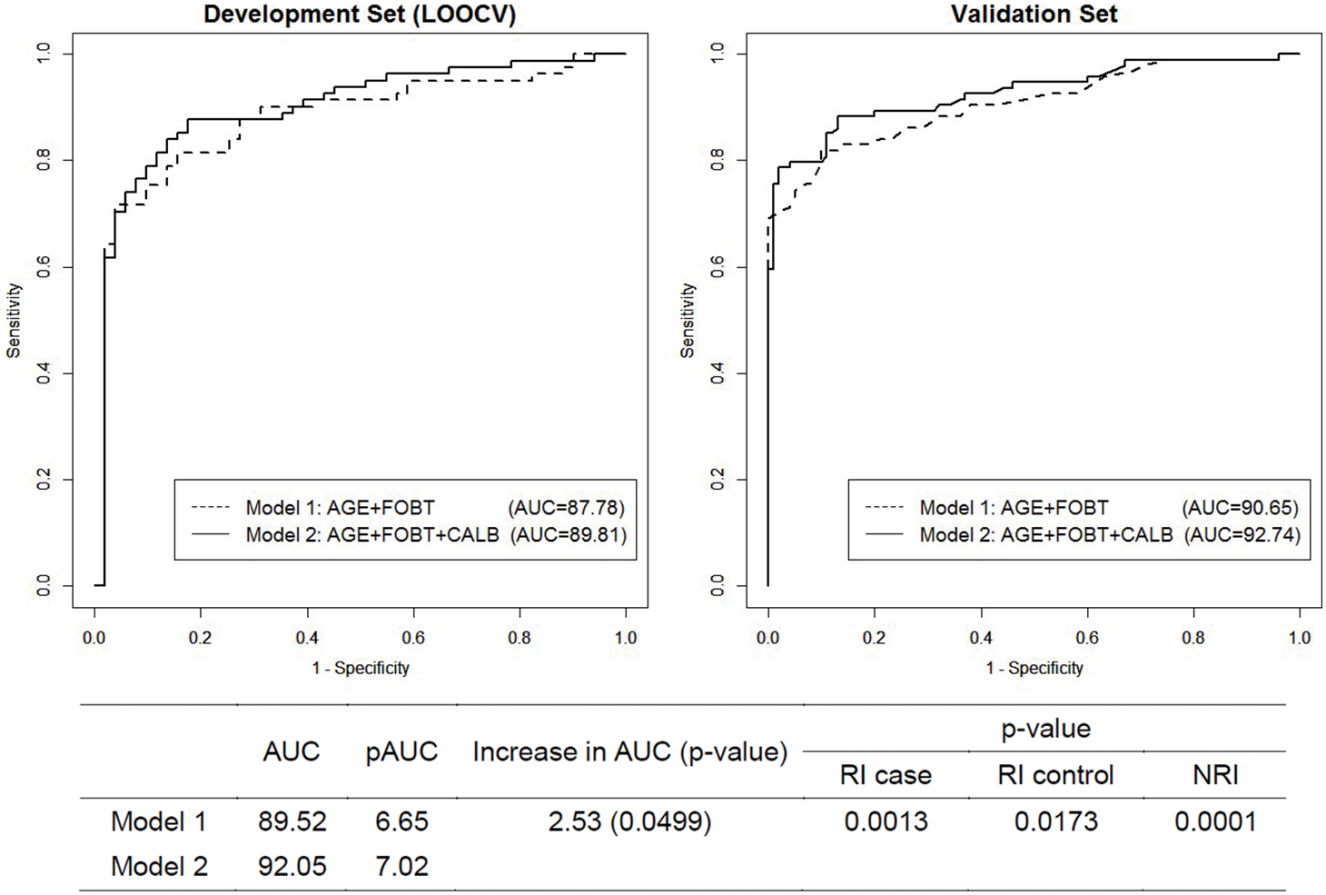
ROC curves for the two models of the development set using leave-one-out cross-validation (LOOCV) and of the validation set.

The incremental benefit of CALB was formally tested by evaluating the increase in AUC [19]. The p-value of the increase in AUC from the model using FOBT alone to the model using both CALB and FOBT was 0.0499 (Figure 2), suggesting that adding CALB to a model that included FOBT significantly improved the AUC. Similarly, the p-values for RI in CRC patients and controls from the model using FOBT alone to the model using both CALB and FOBT were 0.0013 and 0.0173, respectively, and the p-value of NRI was 0.0001. All of these findings indicate that the addition of CALB to the model resulted in a statistically significant improvement in reclassification.

**Figure 3:**
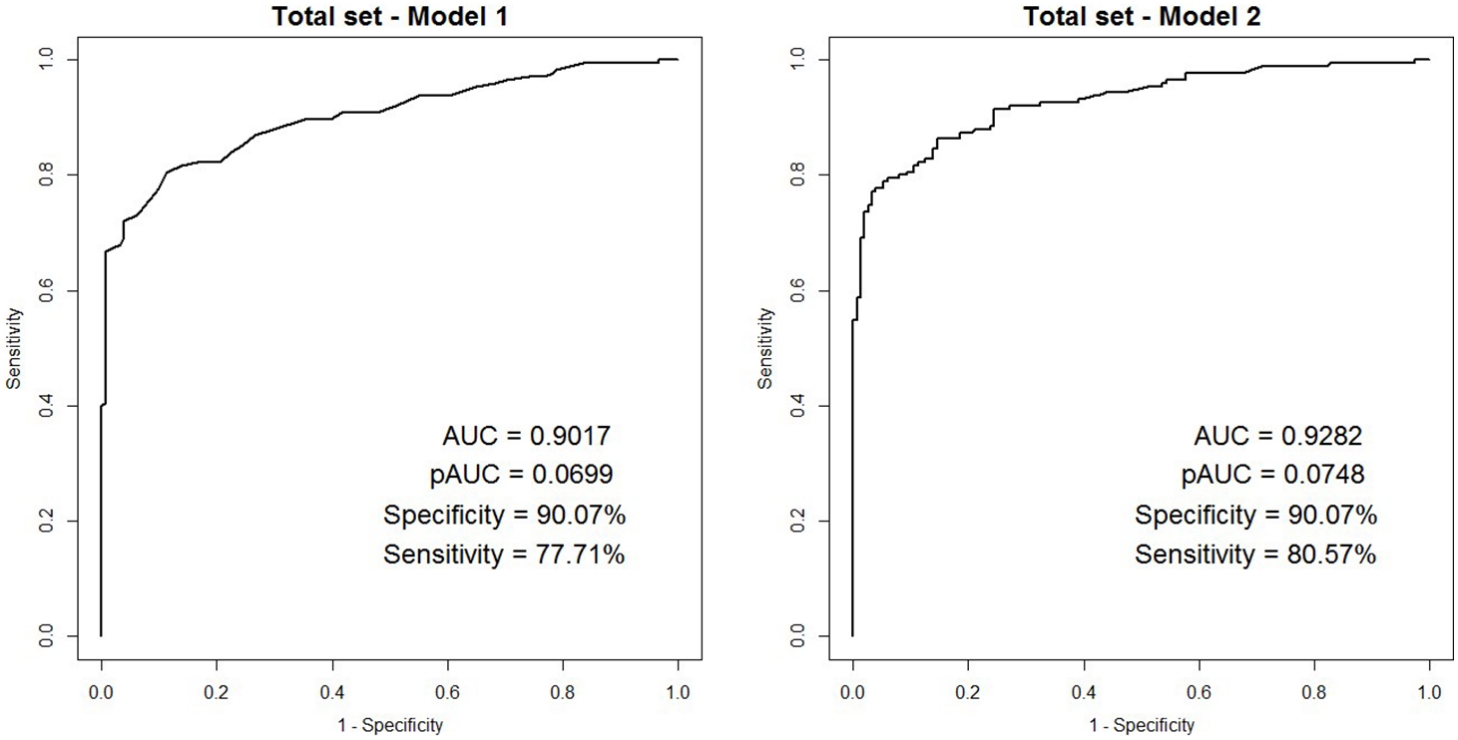
ROC curves for the two models for all patients (total set).

The final model was then fit to the entire data set, which included both the development and validation data sets, to increase its accuracy. ROC, AUC, pAUC, and sensitivity at the specificity closest to 90% are presented in Figure 3 for the models that included FOBT alone and FOBT plus CALB. The model of FOBT alone had an AUC of 92.82% (95% CI 90.05%–95.58%), a pAUC of 7.48%, and a sensitivity of 80.57% at the specificity closest to 90%. The final model consisted of the following equation to predict the probability of colorectal cancer:
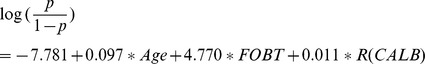
Here, *R(CALB)* is a rank transformed value, making it a relative measure. The rank values of CALB that can be used as inputs for this equation are presented in Table S1. For example, if a person has a CALB value of 2224, the value of *R*(CALB) in the equation is 80. For values lying between two CALB measures, a closest or linear interpolated rank can be used.

## Discussion

FOBT is a conventionally used reference fecal screening test. We found that, at high specificity (90.2%), FOBT alone had only moderate sensitivity (75.31%) for CRC. In evaluating the additive diagnostic accuracy of CALB, a novel fecal diagnostic marker identified in our previous study [7], we found that, at the same specificity (90.2%), the sensitivity of FOBT + CALB for CRC was higher (82.72%). Thus, at high specificity, the combination of the two fecal markers increased the sensitivity of CRC detection.

The diagnostic accuracy of FOBT for CRC in the present study was similar to that reported earlier [9], [20]. In one study, the sensitivity of FOBT in asymptomatic non-referred populations was 26% [21], whereas another study, involving large numbers of patients evaluated by FOBT, reported a sensitivity of 81% [22]. About 80–90% of patients with fecal blood loss >20 ml/day were positive on the FOBT, and the sensitivity of the FOBT correlated with fluctuations in blood loss and intermittent tumor bleeding [9]. These patterns of fecal bleeding may explain the higher positivity rate in T2, T3, and T4 than in T1 cancers and suggest that more advanced tumors bleed more consistently and to a greater extent, improving the diagnostic sensitivity of FOBT in patients with advanced tumors [9]. In contrast, another study found that mean daily blood loss was not affected by tumor stage, but was associated with tumor site, as daily blood loss was lower in patients with left than right sided CRC [23]. Nevertheless, our results, along with those previously reported [9], [23], indicate that the sensitivity of FOBT for CRC increases with advancing cancer stage, with the FOBT positivity rate being lower during early than later stages of cancer. However, CALB showed similar positivity rates, even when comparing early and late stage tumors. Therefore, evaluation of CALB may compensate for the low FOBT positivity rate in early stages of CRC.

We previously reported that CALB was a candidate fecal marker for the diagnosis of CRC [7]. CALB is a component of calprotectin (S100A8/S100A9), which has been used as a fecal marker for IBD and colorectal neoplasms [9], [24], [25]. CALB is secreted by intestinal monocytes and epithelial cells, and is associated with inflammatory processes, including IBD severity [8], [11]. Interestingly, the sensitivity of fecal CALB for CRC was higher than that of FOBT alone (72.0% vs. 62.3%), although the specificity was slightly lower in this study and our earlier study (77.1% vs. 98.7%) [7]. Several control subjects showed false positive results for CALB. These patients may have had a functional intestinal disease, such as irritable bowel syndrome without gross inflammation. Calprotectin, a heterodimer of calgranulin A and calgranulin B, may be elevated in patients with irritable bowel syndrome [26]–[28]. Since individual fecal markers could not perfectly discriminate CRC patients from healthy controls, we tested a combination of two fecal markers.

A combination test that included three fecal markers, tissue inhibitor of metalloproteinase-1 (TIMP-1), CALB, and hemoglobin-haptoglobin, was better at detecting CRC than FOBT alone [29]. Furthermore, a fecal assay for cyclooxygenase-2 and matrix metalloproteinase 7 mRNAs may be a promising screening test for CRC [30]. A concept similar to ours was applied in ovarian cancer, with results showing that a combination of four serologic markers (leptin, prolactin, osteopontin, and insulin-like growth factor-II) had greater sensitivity and specificity for ovarian cancer than any of these markers alone [31]. The incremental benefit of a new marker in risk prediction can be evaluated by analyzing the increase in AUC. In the absence of a high degree of association with the new marker, however, AUC is unlikely to increase significantly [32]–[34]. Despite this difficulty, adding CALB to the model meaningfully improved its predictive ability, as shown by the significant increase in AUC. As an alternative to increased AUC, we used NRI [18] to quantify the improvement in classification resulting from the use of a model with a new marker. RI was first evaluated separately in CRC patients and controls, and their combination (NRI) was assessed. The p-values for RI in patients and controls from model 1 to model 2 were 0.001 and 0.017, respectively and the p-value for NRI was 0.0001, indicating that the inclusion of the fecal CALB test resulted in a statistically significant improvement in reclassification for both patients and controls. These results indicated that the predictive ability of the model with FOBT alone may be significantly improved by the addition of CALB.

This study had several limitations. First, the number of subjects in each group was relatively small, while only 151 healthy controls were included, indicating the need to validate the findings in larger numbers of patients. Second, we enrolled patients with CRC without taking into account symptoms such as hematochezia, constipation and melena. Since screening tests are usually performed in asymptomatic stages, our results require further validation in asymptomatic individuals. Third, the ages of our CRC and control groups differed significantly. However, our predictive model adjusted for age. Finally, although the combination of CALB and FOBT may be more sensitive and specific for CRC than FOBT alone, the sensitivity and specificity of the combination may not be high enough when compared with colonoscopy. Our prediction model, however, might be useful in situations where colonoscopy is unavailable, including in areas without a clinic, hospital, or trained personnel. Also, the proposed prediction model based on stool markers may be helpful and convenient in reducing costs and bowel preparation.

Therefore additional fecal markers may be needed to increase the specificity and sensitivity of fecal screening for CRC.

In conclusion, a combined analysis of two fecal markers, CALB and FOBT, may have greater sensitivity and specificity for CRC than FOBT alone. Further validations are needed to confirm the clinical utility of this combination screening test for CRC.

## Supporting Information

Figure S1 **Schematic of the statistical prediction model through the development and validation data sets.** The model was first fit to the data from the development dataset, followed by internal validation using the leave-one-out cross-validation (LOOCV) method. LOOCV performance was examined, and the model was externally validated on the validation dataset. After acceptable internal and external validations, the final predictive model for use in future subjects was developed using the total dataset, which included both the development and validation datasets, since the accuracy of estimates of the effect of risk factors increases as datasets become larger. In each of the above steps, two models were considered, the first using FOBT alone and the second including both FOBT and CALB. Because there was an imbalance in age between patients and controls, age was adjusted for in both models. Model performance was evaluated by receiver operating curve (ROC) analysis, followed by calculations of the area under the ROC curve (AUC) and the partial area under the ROC curve (pAUC) corresponding to a specificity >0.9. In LOOCV, one sample was set aside (testing) and the predictive model was fit to the remaining samples (training). Based on this prediction model, the probability of CRC in one sample not used in model development (test sample) was estimated. Moreover, the cutoff for predicted probability corresponding to a specificity of 90% was selected, followed by the prediction of whether the testing sample was positive or negative for CRC. This procedure was repeated for a number of times equal to the number of samples in the data set, so that all samples served as a testing sample exactly once. The cross-validated sensitivity was then determined for the specificity closest to 90%, and a cross-validated ROC curve was generated.
(TIF)

Table S1 **Optical density of calgranulin B and the corresponding rank.**
(DOCX)
