# Supplementary figures and images for: A Predictive Model Combining Fecal Calgranulin B and Fecal Occult Blood Tests Can Improve the Diagnosis of Colorectal Cancer
Source: PLoS One. 2014 Sep 4;9(9):e106182. doi: 10.1371/journal.pone.0106182 (PMC4154865; doi:10.1371/journal.pone.0106182)

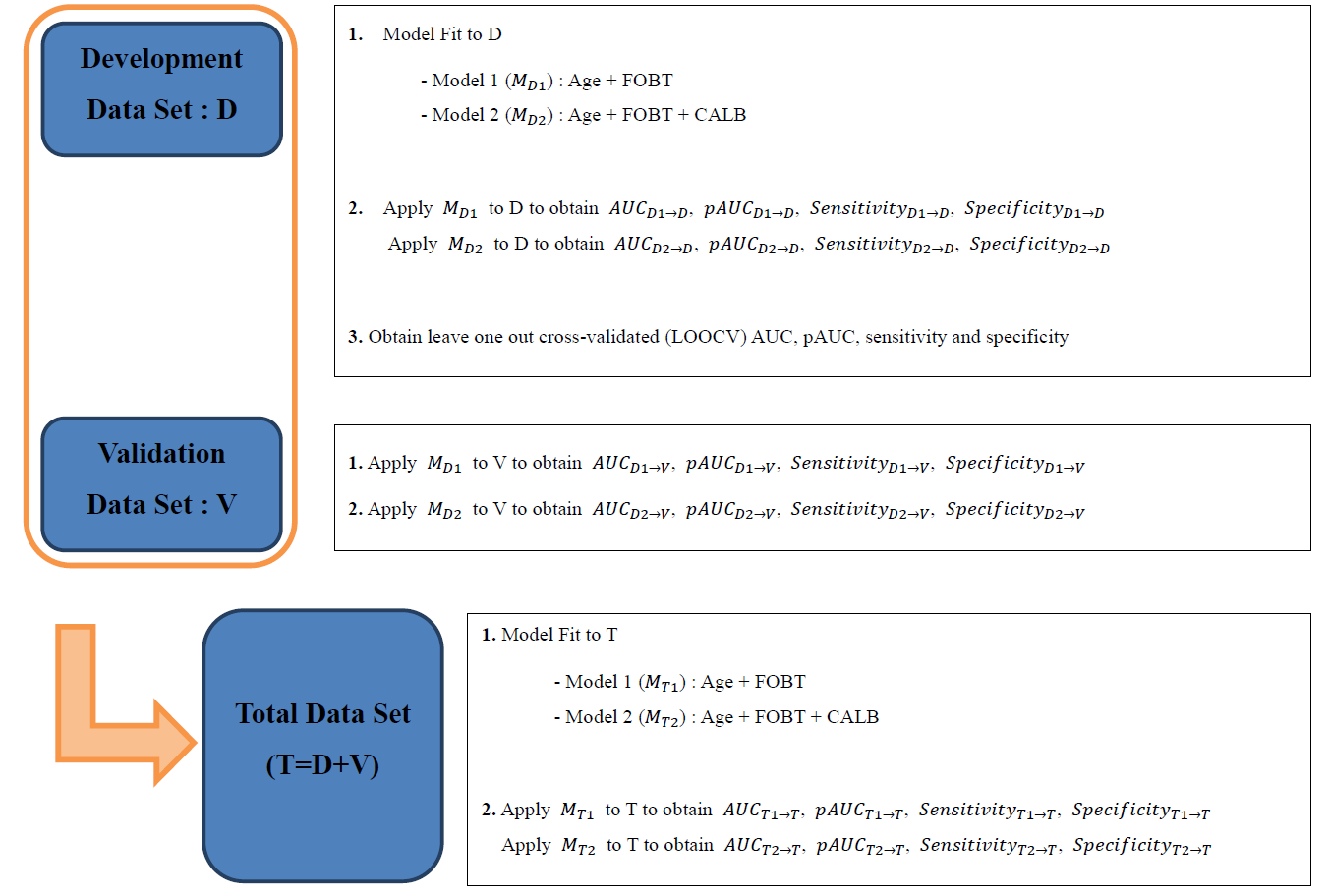

Supplement: Figure S1 — Schematic of the statistical prediction model through the development and validation data sets. The model was first fit to the data from the development dataset, followed by internal validation using the leave-one-out cross-validation (LOOCV) method. LOOCV performance was examined, and the model was externally validated on the validation dataset. After acceptable internal and external validations, the final predictive model for use in future subjects was developed using the total dataset, which included both the development and validation datasets, since the accuracy of estimates of the effect of risk factors increases as datasets become larger. In each of the above steps, two models were considered, the first using FOBT alone and the second including both FOBT and CALB. Because there was an imbalance in age between patients and controls, age was adjusted for in both models. Model performance was evaluated by receiver operating curve (ROC) analysis, followed by calculations of the area under the ROC curve (AUC) and the partial area under the ROC curve (pAUC) corresponding to a specificity >0.9. In LOOCV, one sample was set aside (testing) and the predictive model was fit to the remaining samples (training). Based on this prediction model, the probability of CRC in one sample not used in model development (test sample) was estimated. Moreover, the cutoff for predicted probability corresponding to a specificity of 90% was selected, followed by the prediction of whether the testing sample was positive or negative for CRC. This procedure was repeated for a number of times equal to the number of samples in the data set, so that all samples served as a testing sample exactly once. The cross-validated sensitivity was then determined for the specificity closest to 90%, and a cross-validated ROC curve was generated. (TIF) [file pone.0106182.s001.tif]
