# Supplementary material for: A Predictive Model Combining Fecal Calgranulin B and Fecal Occult Blood Tests Can Improve the Diagnosis of Colorectal Cancer
Source: PLoS One. 2014 Sep 4;9(9):e106182. doi: 10.1371/journal.pone.0106182 (PMC4154865; doi:10.1371/journal.pone.0106182)
Supplement: Table S1 — Optical density of calgranulin B and the corresponding rank. (DOCX) [file pone.0106182.s002.docx]

**Table S1. Optical density of calgranulin B and the corresponding rank**

| **CALB** | **CALB rank** | **CALB** | **CALB rank** | **CALB** | **CALB rank** | **CALB** | **CALB rank** |
| --- | --- | --- | --- | --- | --- | --- | --- |
| 0 | 62.5 | 2162.99 | 175 | 2861.76 | 226 | 3571.51 | 278 |
| 1325.04 | 125 | 2192.11 | 176 | 2874.94 | 227 | 3586.61 | 279 |
| 1337.69 | 126 | 2192.64 | 177 | 2879.68 | 228 | 3596.42 | 280 |
| 1409.29 | 127 | 2207.45 | 178 | 2879.88 | 229 | 3607.4 | 281 |
| 1443.77 | 128 | 2209.51 | 179 | 2883.8 | 230 | 3619.28 | 282 |
| 1471.56 | 129 | 2224 | 180 | 2892.04 | 231 | 3625.98 | 283 |
| 1492.36 | 130 | 2251.25 | 181 | 2896.32 | 232 | 3641.82 | 284 |
| 1507.28 | 131 | 2253.92 | 182 | 2942.37 | 233 | 3677.19 | 285 |
| 1524.75 | 132 | 2266.64 | 183 | 2957.62 | 234 | 3692.29 | 286 |
| 1560.75 | 133 | 2274.08 | 184 | 2963.41 | 235 | 3700.9 | 287 |
| 1562.05 | 134 | 2307.67 | 185 | 2967.59 | 236 | 3738.22 | 288 |
| 1598.27 | 135 | 2316.2 | 186 | 2969.71 | 237 | 3748.39 | 289 |
| 1610.92 | 136 | 2317.07 | 187 | 2992.54 | 238 | 3793.02 | 290 |
| 1614.46 | 137 | 2329.31 | 188 | 3015.08 | 239 | 3795.06 | 291 |
| 1616.16 | 138 | 2330.84 | 189 | 3038.96 | 240 | 3816.53 | 292 |
| 1631.94 | 139 | 2354.54 | 190 | 3052.86 | 241 | 3821.72 | 293 |
| 1644.04 | 140 | 2375.95 | 191 | 3058.51 | 242 | 3870.31 | 294 |
| 1670.76 | 141 | 2409.8 | 192 | 3069.85 | 243 | 3911.31 | 295 |
| 1673.77 | 142 | 2430.33 | 193 | 3081.51 | 244 | 3955.33 | 296 |
| 1707.42 | 143 | 2437.34 | 194 | 3092.87 | 245 | 3980.4 | 297 |
| 1716.65 | 144 | 2439.51 | 195 | 3124.23 | 246 | 3985.85 | 298 |
| 1721.34 | 145 | 2440.51 | 196 | 3125.03 | 247 | 4006.44 | 299 |
| 1726.12 | 146 | 2477.13 | 197 | 3127.61 | 248 | 4029.57 | 300 |
| 1733.35 | 147 | 2480.98 | 198 | 3167.83 | 249 | 4140.58 | 301 |
| 1743.53 | 148 | 2484.78 | 199 | 3171.87 | 250 | 4150.31 | 302 |
| 1747.4 | 149 | 2486.49 | 200 | 3173.79 | 251 | 4190.3 | 303 |
| 1782.19 | 150 | 2487.51 | 201 | 3178.89 | 252 | 4235.94 | 304 |
| 1783.15 | 151 | 2526.96 | 202 | 3188.37 | 253 | 4285.65 | 305 |
| 1783.34 | 152 | 2557.47 | 203 | 3203.87 | 254 | 4323.81 | 306 |
| 1800.26 | 153 | 2566.77 | 204 | 3205.94 | 255 | 4361.81 | 307 |
| 1814.21 | 154 | 2568.95 | 205 | 3209.02 | 256 | 4417.94 | 308 |
| 1859.49 | 155 | 2580.48 | 206 | 3228.02 | 257 | 4426.74 | 309 |
| 1875.85 | 156 | 2605.62 | 207 | 3254.38 | 258 | 4448.98 | 310 |
| 1879.05 | 157 | 2605.88 | 208 | 3296.15 | 259 | 4585.7 | 311 |
| 1884.39 | 158 | 2628.75 | 209 | 3300.29 | 260 | 4591.56 | 312 |
| 1884.96 | 159 | 2631.81 | 210 | 3304.34 | 261 | 4608.09 | 313 |
| 1892.98 | 160 | 2634.72 | 211 | 3325.18 | 262 | 4692.92 | 314 |
| 1913.92 | 161 | 2638.07 | 212 | 3339.34 | 263 | 4731.09 | 315 |
| 1920.47 | 162 | 2661.05 | 213 | 3340.59 | 264 | 4787.83 | 316 |
| 1995.71 | 163 | 2691.79 | 214 | 3359.48 | 265 | 4793.19 | 317 |
| 1998.79 | 164 | 2693 | 215 | 3361.72 | 266 | 4804.9 | 318 |
| 2006.12 | 165 | 2698.07 | 216 | 3363.79 | 267 | 4862.87 | 319 |
| 2014.78 | 166 | 2708.39 | 217 | 3373.83 | 268 | 4926.25 | 320 |
| 2044.95 | 167 | 2713.9 | 218 | 3374.09 | 269.5 | 4927.22 | 321 |
| 2054.03 | 168 | 2754.31 | 219 | 3378.28 | 271 | 4960.48 | 322 |
| 2075.4 | 169 | 2766.64 | 220 | 3410.34 | 272 | 5160.61 | 323 |
| 2100.31 | 170 | 2779.13 | 221 | 3411.19 | 273 | 5181.16 | 324 |
| 2150.37 | 171 | 2830.97 | 222 | 3466.87 | 274 | 5288.79 | 325 |
| 2156.39 | 172 | 2831.66 | 223 | 3496.7 | 275 | 5432.08 | 326 |
| 2160.71 | 173 | 2842.15 | 224 | 3508.39 | 276 |  |  |
| 2162.21 | 174 | 2845.45 | 225 | 3508.5 | 277 |  |  |
